# Supplementary material for: GREIN: An Interactive Web Platform for Re-analyzing GEO RNA-seq Data
Source: Sci Rep. 2019 May 20;9:7580. doi: 10.1038/s41598-019-43935-8 (PMC6527554; doi:10.1038/s41598-019-43935-8)
Supplement: Supplementary file 1 — Supplementary Information [file 41598_2019_43935_MOESM1_ESM.pdf]

GREIN: An Interactive Web Platform for Re-analyzing GEO RNA-seq Data

Naim Al Mahi[1], Mehdi Fazel Najafabadi[1], Marcin Pilarczyk[1], Michal Kouril[2], and Mario Medvedovic[1,*]

[1]Division of Biostatistics and Bioinformatics, Department of Environmental Health, University of Cincinnati, 3223 Eden Avenue, Cincinnati, OH 45220, USA
[2]Division of Biomedical Informatics, Cincinnati Children's Hospital Medical Center, Cincinnati, OH, USA

Supplementary Figures

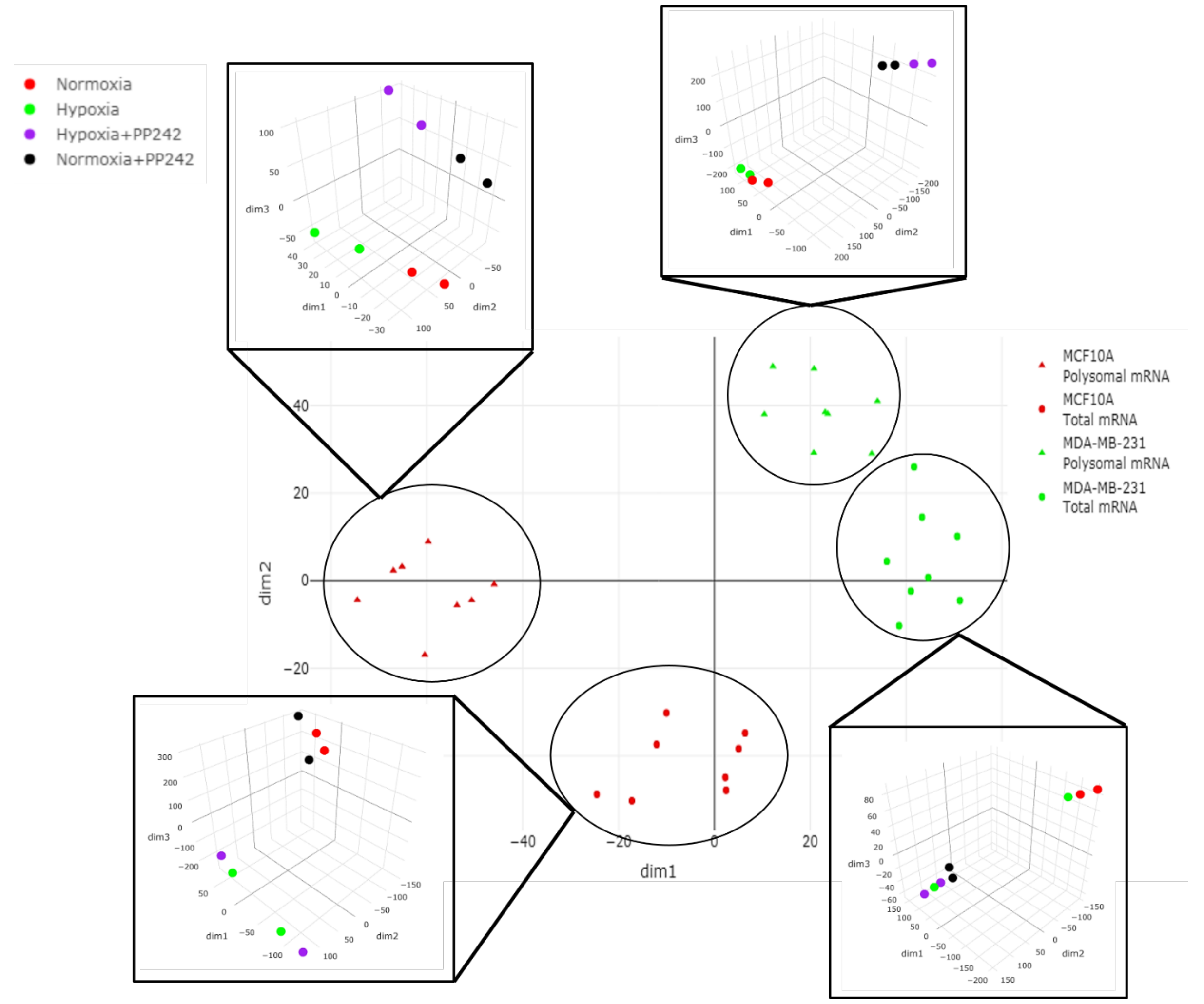

Supplementary Figure S1. 2D t-SNE plot of cell line and mRNA fraction along with 3D t-SNE sub-plots of experimental conditions. A clear separation exist between the cell lines and mRNA fractionation is also distinguishable within each cell line. Furthermore, 3D t-SNE sub-plots of the experimental conditions within each of these fractions and cell lines represent inherent variability between the conditions.

Supplementary Tables

Tool	Purpose	Version
1. RNASeqPower[1]	Power analysis	1.18.0
2. Shiny[2]	GREIN GUI	1.0.5
3. GEOquery[3]	Metadata	2.46.14
4. edgeR[4]	Differential expression	3.20.8
5. Plotly[5]	Exploratory plots	4.7.1
6. ComplexHeatmap[6]	Static heatmap	1.17.1
7. Iheatmapr[7]	Interactive heatmap	0.4.3
8. Rgl[8]	PCA	0.99.9
9. Rtsne[9]	t-SNE	0.13
10. Aspera connect[10]	Download raw data	3.7.2
11. SRA toolkit[11]	Download SRA files	2.8.2
12. FastQC[12]	Quality control report of the fastq files	0.11.4
13. Trimmomatic[13]	Trim fastq files	0.36
14. Salmon[14]	Read mapping	0.8.2
15. Tximport[15]	Convert transcript level estimates to gene level	1.6.0
16. MultiQC[16]	Combined report of fastq files and read mapping	1.2

Supplementary Table S1. List of tools included in GREIN and GREP2. Tools from 1 to 9 are used in GREIN and the rest in GREP2.

Tool	Category	ID	Name	FDR (B&H)
ToppGene	GO: Biological Process	GO:0071456	cellular response to hypoxia	2.53E-07
ToppGene	GO: Biological Process	GO:0036294	cellular response to decreased oxygen levels	2.53E-07
ToppGene	GO: Biological Process	GO:0071453	cellular response to oxygen levels	2.57E-07
ToppGene	GO: Biological Process	GO:0001666	response to hypoxia	2.57E-07
ToppGene	GO: Biological Process	GO:0036293	response to decreased oxygen levels	2.98E-07
ToppGene	GO: Biological Process	GO:0070482	response to oxygen levels	4.85E-07
ToppGene	GO: Biological Process	GO:0009628	response to abiotic stimulus	1.17E-06
ToppGene	GO: Biological Process	GO:0018401	peptidyl-proline hydroxylation to 4-hydroxy-L-proline	7.42E-04
ToppGene	GO: Biological Process	GO:0061621	canonical glycolysis	1.80E-03
ToppGene	GO: Biological Process	GO:0061718	glucose catabolic process to pyruvate	1.80E-03
DAVID	GOTERM_BP_DIRECT	GO:0071456	cellular response to hypoxia	1.91E-05
DAVID	GOTERM_BP_DIRECT	GO:0030855	epithelial cell differentiation	1.84E-04
DAVID	GOTERM_BP_DIRECT	GO:0001666	response to hypoxia	2.23E-04
DAVID	GOTERM_BP_DIRECT	GO:0003007	heart morphogenesis	0.006402
DAVID	GOTERM_BP_DIRECT	GO:0002576	platelet degranulation	0.007785
DAVID	GOTERM_BP_DIRECT	GO:0030199	collagen fibril organization	0.011101
DAVID	GOTERM_BP_DIRECT	GO:0001525	angiogenesis	0.017601
DAVID	GOTERM_BP_DIRECT	GO:0051781	positive regulation of cell division	0.018381
DAVID	GOTERM_BP_DIRECT	GO:0042462	eye photoreceptor cell development	0.019052
DAVID	GOTERM_BP_DIRECT	GO:0042127	regulation of cell proliferation	0.023541

Supplementary Table S2. Top 10 GO: Biological processes in MCF10A cell line from ToppGene and DAVID functional annotation tool. Differentially expressed and detectable genes from the comparison between hypoxia and normoxia in MCF10A cell line with total mRNA fractionation are uploaded in iLINCS and analyzed via ToppGene suite.

Tool	Category	ID	Name	FDR (B&H)
ToppGene	GO: Biological Process	GO:0070482	response to oxygen levels	9.28E-13
ToppGene	GO: Biological Process	GO:0001666	response to hypoxia	1.96E-12
ToppGene	GO: Biological Process	GO:0036293	response to decreased oxygen levels	2.14E-12
ToppGene	GO: Biological Process	GO:0055114	oxidation-reduction process	3.21E-10
ToppGene	GO: Biological Process	GO:0009628	response to abiotic stimulus	2.01E-08
ToppGene	GO: Biological Process	GO:0018126	protein hydroxylation	4.72E-08
ToppGene	GO: Biological Process	GO:0046031	ADP metabolic process	4.72E-08
ToppGene	GO: Biological Process	GO:0032787	monocarboxylic acid metabolic process	4.87E-08
ToppGene	GO: Biological Process	GO:0006096	glycolytic process	1.29E-07
ToppGene	GO: Biological Process	GO:0009179	purine ribonucleoside diphosphate metabolic process	1.42E-07
DAVID	GOTERM_BP_DIRECT	GO:0001666	response to hypoxia	1.03E-15
DAVID	GOTERM_BP_DIRECT	GO:0098609	cell-cell adhesion	1.81E-08
DAVID	GOTERM_BP_DIRECT	GO:0001525	angiogenesis	1.67E-05
DAVID	GOTERM_BP_DIRECT	GO:0042127	regulation of cell proliferation	1.78E-04
DAVID	GOTERM_BP_DIRECT	GO:0008284	positive regulation of cell proliferation	2.35E-04
DAVID	GOTERM_BP_DIRECT	GO:0030335	positive regulation of cell migration	5.29E-04
DAVID	GOTERM_BP_DIRECT	GO:0042327	positive regulation of phosphorylation	8.28E-04
DAVID	GOTERM_BP_DIRECT	GO:0000188	inactivation of MAPK activity	8.28E-04
DAVID	GOTERM_BP_DIRECT	GO:0030198	extracellular matrix organization	1.02E-03
DAVID	GOTERM_BP_DIRECT	GO:0032570	response to progesterone	1.08E-03

Supplementary Table S3. Top 10 GO: Biological processes in MDA-MB-231 cell line from ToppGene and DAVID functional annotation tool. Differentially expressed and detectable genes from the comparison between hypoxia and normoxia in MDA-MB-231 cell line with total mRNA fractionation are uploaded in iLINCS.

Category	ID	Name	Database	FDR (B&H)
Pathway	138045	HIF-1-alpha transcription factor network	BioSystems: Pathway Interaction Database	2.18E-09
Pathway	1270429	DNA Damage/Telomere Stress Induced Senescence	BioSystems: REACTOME	6.24E-06
Pathway	1270426	Cellular Senescence	BioSystems: REACTOME	4.12E-05
Pathway	1270431	Senescence-Associated Secretory Phenotype (SASP)	BioSystems: REACTOME	4.12E-05
Pathway	1269867	Meiotic synapsis	BioSystems: REACTOME	5.22E-05
Pathway	1269864	Packaging of Telomere Ends	BioSystems: REACTOME	5.82E-05
Pathway	1339140	Activation of anterior HOX genes in hindbrain development during early embryogenesis	BioSystems: REACTOME	5.82E-05
Pathway	1339139	Activation of HOX genes during differentiation	BioSystems: REACTOME	5.82E-05
Pathway	137956	HIF-2-alpha transcription factor network	BioSystems: Pathway Interaction Database	1.09E-04
Pathway	1270414	Cellular responses to stress	BioSystems: REACTOME	1.13E-04

Supplementary Table S4. Top 10 pathways from activated in MCF10A. Differentially expressed and detectable genes from the comparison between hypoxia and normoxia in MCF10A cell line with mRNA fractionation are uploaded in iLINCS and analyzed via ToppGene suite.

Category	ID	Name	Database	FDR (B&H)
Pathway	138045	HIF-1-alpha transcription factor network	BioSystems: Pathway Interaction Database	5.06E-15
Pathway	695200	HIF-1 signaling pathway	BioSystems: KEGG	1.18E-09
Pathway	M3468	Genes encoding enzymes and their regulators involved in the remodeling of the extracellular matrix	MSigDB C2 BIOCARTA (v6.0)	6.47E-07
Pathway	1269959	Glucose metabolism	BioSystems: REACTOME	3.44E-05
Pathway	1270245	Collagen formation	BioSystems: REACTOME	7.15E-05
Pathway	1269960	Glycolysis	BioSystems: REACTOME	7.28E-05
Pathway	PW:0000640	glycolysis pathway	Pathway Ontology	4.01E-04
Pathway	M5885	Ensemble of genes encoding ECM-associated proteins including ECM-affilaited proteins, ECM regulators and secreted factors	MSigDB C2 BIOCARTA (v6.0)	4.47E-04
Pathway	PW:0000243	vascular endothelial growth factor signaling	Pathway Ontology	5.21E-04
Pathway	MAP00010	MAP00010 Glycolysis Gluconeogenesis	GenMAPP	5.21E-04

Supplementary Table S5. Top 10 pathways activated in MDA-MB-231. Differentially expressed and detectable genes from the comparison between hypoxia and normoxia in MDA-MB-231 cell line with mRNA fractionation are uploaded in iLINCS and analyzed via ToppGene suite.

Pathway ID	Database	Name	DE&DT target genes	FDR (B&H)
1268855	BioSystems: REACTOME	Diseases of signal transduction	TGFBR2*, PSMD1*, FGF2*, PSMB2, NF1, PSMB1, KRAS, POLR2I, HDAC3, POLR2A, ARAF, FGFR1, APC	3.09E-03
83105	BioSystems: KEGG	Pathways in cancer	TGFBR2*, CUL2, NFKBIA*, FGF2*, KRAS, ARAF, VHL, RXRA, FGFR1, APC, NCOA4 , ARNT	3.09E-03
1268854	BioSystems: REACTOME	Disease	XRCC5*, TGFBR2*, PSMD1* , NFKBIA*, CALR*, FGF2*, PSMB2, NF1, PSMB1, KRAS, POLR2I, HDAC3, POLR2A, ARAF, FGFR1, APC, RPS19, RPS6, CDK5	4.58E-03
1270415	BioSystems: REACTOME	Cellular response to hypoxia	PSMD1*, CUL2, PSMB2, PSMB1, VHL, ARNT	4.58E-03
1270416	BioSystems: REACTOME	Regulation of Hypoxia-inducible Factor (HIF) by oxygen	PSMD1*, CUL2, PSMB2, PSMB1, VHL, ARNT	4.58E-03
1269428	BioSystems: REACTOME	Signaling by Insulin receptor	PSMD1*, PIK3R4, FGF2*, PSMB2, ATP6V0B, NF1, PSMB1, KRAS, ARAF, FGFR1, RPS6	4.58E-03
1269431	BioSystems: REACTOME	IRS-mediated signaling	PSMD1*, PIK3R4, FGF2*, PSMB2, NF1, PSMB1, KRAS, ARAF, FGFR1, RPS6	6.42E-03
1269429	BioSystems: REACTOME	Insulin receptor signaling cascade	PSMD1*, PIK3R4, FGF2*, PSMB2, NF1, PSMB1, KRAS, ARAF, FGFR1, RPS6	6.42E-03
1269690	BioSystems: REACTOME	mRNA Splicing - Major Pathway	SNRPD1*, LSM6, LSM5, CHERP, POLR2I, POLR2A, PRPF6, SRSF3	6.42E-03
1269620	BioSystems: REACTOME	IRS-related events triggered by IGF1R	PSMD1*, PIK3R4, FGF2*, PSMB2, NF1, PSMB1, KRAS, ARAF, FGFR1, RPS6	6.42E-03

Supplementary Table S6. Top 10 pathways activated and target genes in MCF10A cell line. A combined list of DE and NDE&DT genes (DE&DT) from the comparison between hypoxia and normoxia in MCF10A cell line with mRNA fractionation are uploaded in iLINCS and compared with LINCS consensus (CGS) gene knockdown signatures. We selected top 100 knockdown signatures most concordant with our uploaded signatures for enrichment analysis. The target genes in the table represent either not differentially expressed (NDE) or not differentially expressed but detectable (NDE&DT*) genes.

Pathway ID	Database	Name	Target genes (NDE or NDE&DT*)	FDR (B&H)
1270415	BioSystems: REACTOME	Cellular response to hypoxia	VHL, UBE2D1, ARNT, CUL2, PSMD1, PSMD4, PSMB2, PSMB1	3.68E-08
1270416	BioSystems: REACTOME	Regulation of Hypoxia-inducible Factor (HIF) by oxygen	VHL, UBE2D1, ARNT, CUL2, PSMD1, PSMD4, PSMB2, PSMB1	3.68E-08
1270418	BioSystems: REACTOME	Oxygen-dependent proline hydroxylation of Hypoxia-inducible Factor Alpha	VHL, UBE2D1, CUL2, P SMD1, PSMD4, PSMB2, PSMB1	1.79E-07
83105	BioSystems: KEGG	Pathways in cancer	MET*, RXRA, RB1, VHL, TGFBR2, RAD51, BIRC5, PLCG1, ARNT, APC, PIAS2, CUL2, SMAD2, ARAF	8.36E-07
83107	BioSystems: KEGG	Renal cell carcinoma	MET*, VHL, ARNT, CUL2, ARAF	4.05E-05
1270414	BioSystems: REACTOME	Cellular responses to stress	RB1, VHL, UBE2D1, MAPKAPK3, SOD1, ARNT, CUL2, TERF2IP, PSMD1, PSMD4, PSMB2, PSMB1	1.76E-04
M13324	MSigDB C2 BIOCARTA (v6.0)	Hypoxia-Inducible Factor in the Cardiovascular System	ASPH*, VHL, ARNT	3.0E-04
138056	BioSystems: Pathway Interaction Database	Hypoxic and oxygen homeostasis regulation of HIF-1-alpha	VHL, ARNT, CUL2	3.0E-04
695200	BioSystems: KEGG	HIF-1 signaling pathway	VHL, PLCG1, ARNT, CUL2, RPS6	4.54E-04
1268855	BioSystems: REACTOME	Diseases of signal transduction	MET*, TGFBR2, PLCG1 , POLR2A, APC, SMAD2, HDAC3, PSMD1, PSMD4, PSMB2, ARAF, PSMB1	4.54E-04

Supplementary Table S7. Top 10 pathways activated and target genes in MDA-MB-231 cell line. A combined list of DE and NDE&DT genes (DE&DT) from the comparison between hypoxia and normoxia in MDA-MB-231cell line with mRNA fractionation are uploaded in iLINCS and compared with LINCS consensus (CGS) gene knockdown signatures. We selected top 100 knockdown signatures most concordant with our uploaded signatures for enrichment analysis. The target genes in the table represent either not differentially expressed (NDE) or not differentially expressed but detectable (NDE&DT*) genes.

Step-by-step guide of GREIN with an example dataset

Landing page (GEO datasets)

To illustrate the usability and efficacy of GREIN, we will walk through the available features for exploring and analyzing data sets with an example. The interpretation of the results need further bioinformatics expertise. GREIN is accessible at: https://shiny.ilincs.org/grein.

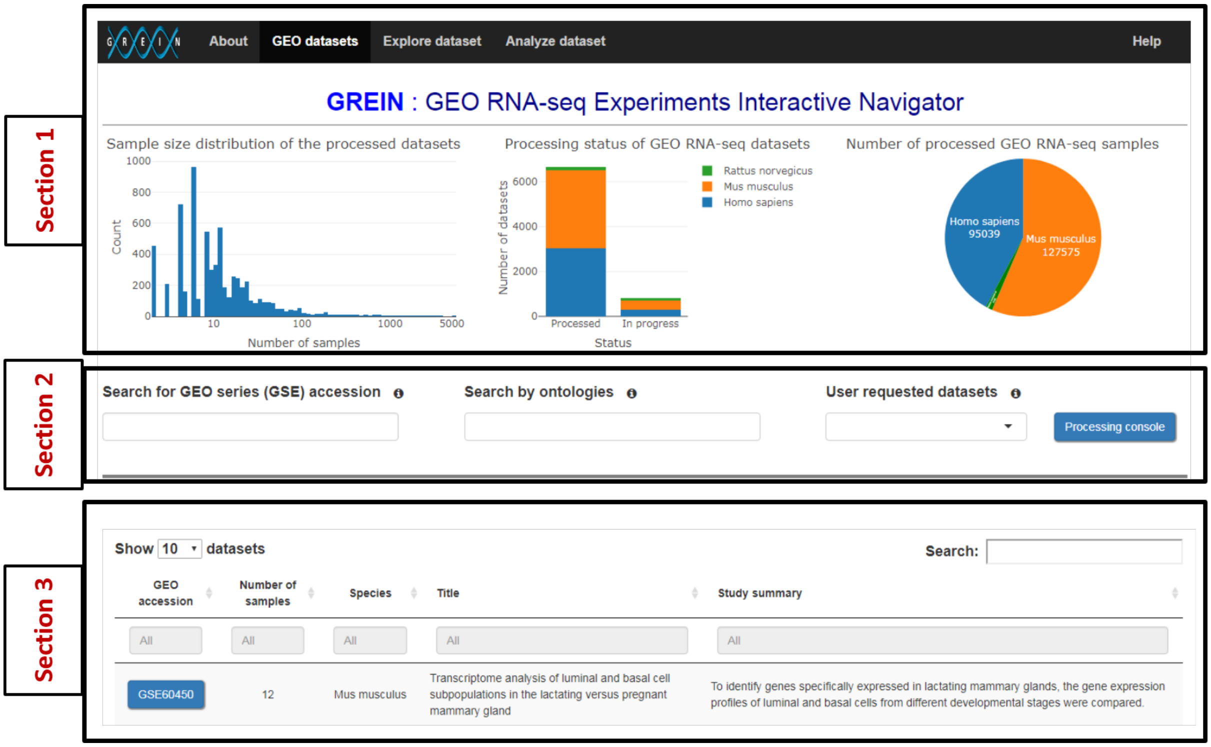

Supplementary Figure S2. GREIN landing page. The first section provides data summaries, second section allows search options along with the list of user requested datasets and their processing status, and the processed datasets are shown in the third section.

Section 1

The first section (Supplementary Figure S2) provides information regarding the sample size distribution of the processed datasets, total number of data sets already processed or waiting to be processed, and the number of processed human, mouse, or rat samples by our GREP2 pipeline.

Section 2

The first panel in section 2 provides the option to process GEO dataset of interest if it is not already processed. GEO RNA-seq datasets can be searched using a GEO series accession to see if it exists in the dataset table (Section 3). If not, then **'Start processing'** button will appear right below this box and the processing can be initialized by clicking this button which opens the **'Processing console'** window (See Supplementary Figure S3). Requested data set id can be seen in the **'Currently processing'** or at the bottom

of the **'Waiting to process'** menu. This window also shows the logs of the currently processing dataset requested by a user. A single server processing pipeline is continuously running in the back-end to process datasets whenever requested. This pipeline is dedicated to process the user requested datasets only. Depending on the size of the data and queue, the requested data sets are automatically uploaded to the portal as soon as they are processed.

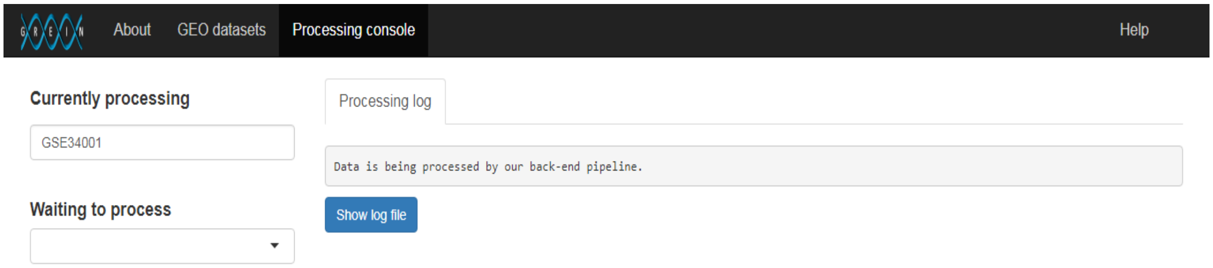

Supplementary Figure S3. Processing console window. Clicking the 'Show log file' button will open the processing log of user requested dataset.

GREIN also provides search options for biomedical ontologies (for example, cancer, basal cell, kidney, etc.) in the second panel of this section. We use ontology terms mapped to GEO samples by MetaSRA project[17] (http://metasra.biostat.wisc.edu/). User search term associated ontologies can be found in the **'Metadata'** under **'Explore dataset'** tab.

The right most panel in this section shows the user requested data sets. If a dataset is requested for processing, the dataset id (GEO series accession) will show up here. Also, the status of the processing queue can be opened by pressing the **'Processing console'** button.

Section 3

The list of processed data sets with additional information in the data table is shown in section 4 (See Supplementary Figure S2). Two types of search options are available in this table. Search box at the top-right of the table lets a user to search anything in this table. Other search boxes at the top of each column enables column-wise searching. User can start exploring a dataset by clicking the GEO accession in the first column.

Explore dataset

Let us demonstrate the features of GREIN for exploring and analyzing an RNA-seq data by searching **'GSE60450'** either at the top-right or first column's search box in the dataset table. Clicking **'GSE60450'**, will open the **'Explore dataset'** tab. Fu *et al.*[18] conducted this experiment to examine the change in expression profiles between luminal and basal cells in mouse mammary glands of virgin, pregnant, and lactating mice.

Description

This tab panel provides descriptive information including study link, number of GEO samples, number of SRA runs, title, and study summary of the corresponding dataset.

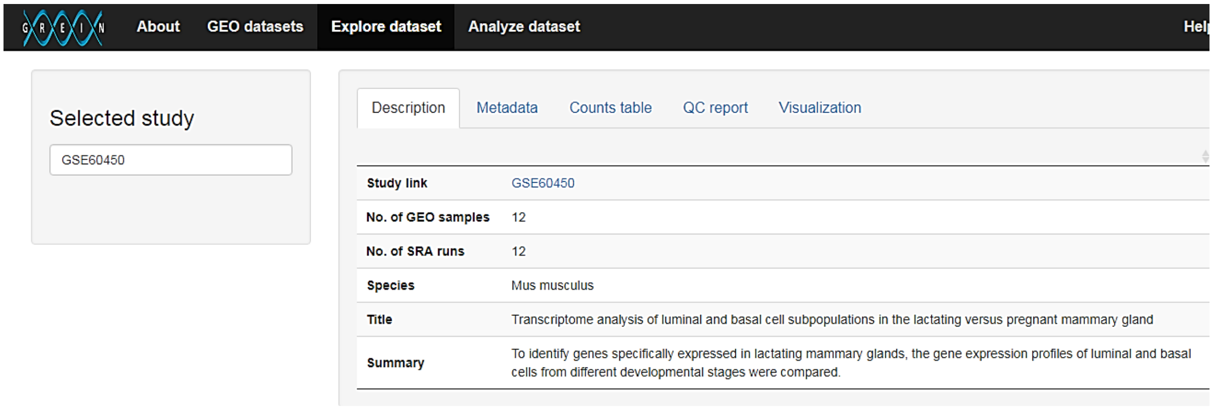

Supplementary Figure 4. Description tab panel. The study link will take the user to GEO web page.

Metadata

GEO metadata contains a lot of information, although not all of these are useful for analysis or visualization purpose. So, we provide a filtered version of the metadata besides the full metadata.

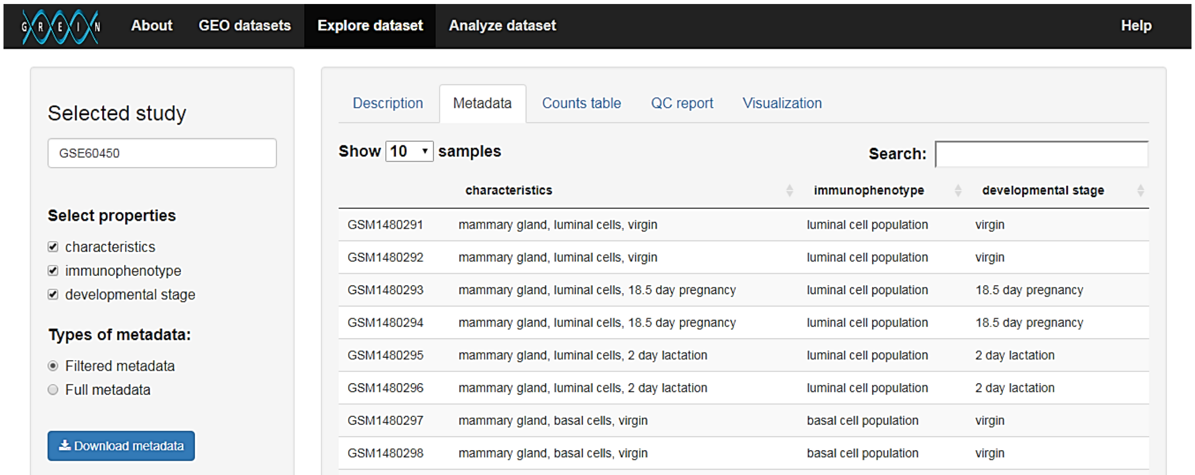

Supplementary Figure S5. Metadata tab panel. Both the full and filtered metadata are downloadable in CSV format.

We filter metadata based on the following criteria:

1. Columns that contain a single value.

2. Columns with incoherent information regarding analysis and visualization such as dates, time, download path and so on.

This dataset (GSE60450) has two cell types and three developmental stages and each combination has two biological replicates. User can also download both the filtered and full metadata.

Counts table

This table shows gene wise estimated read abundance (rounded to the nearest integer) for each sample both in raw and normalized format. We use *Salmon* to quantify transcript abundances for each sample. These transcript level estimates are then summarized to gene level using Bioconductor package *tximport* which gives estimated counts scaled up to library size while taking into account for transcript length. We obtained gene annotation for Homo sapiens (GRCh38), Mus musculus (GRCm38), and Rattus norvegicus (Rnor_6.0) from Ensemble (release-91). Both gene and transcript level expression data are downloadable. Also, each gene can be visualized via interactive boxplots.

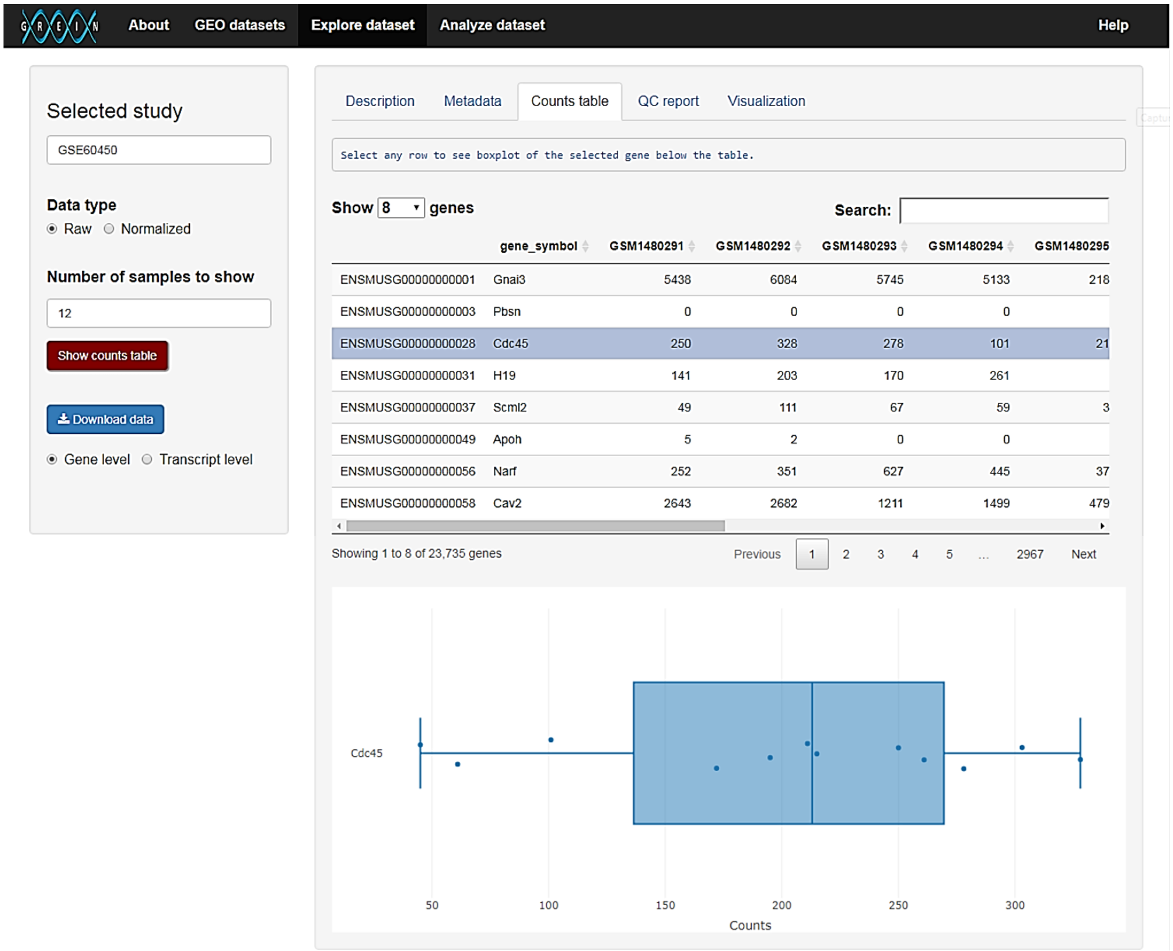

Supplementary Figure S6. Counts table tab panel. Both the gene and transcript level counts are downloadable in CSV format. User can change the number of samples or genes to show. A global search option is also available. Gene expression pattern can be visualized via boxplot.

Quality control (QC) report

After running *FastQC* and *Salmon*, we generate a combined quality control report of all the samples using *MultiQC*. This downloadable report contains information regarding read mapping and quality scores of the FastQ files. In the general statistics table, each sample corresponds to two rows, the first one for the Salmon read mapping and the second one for *FastQC* (See Supplementary Figure S7).

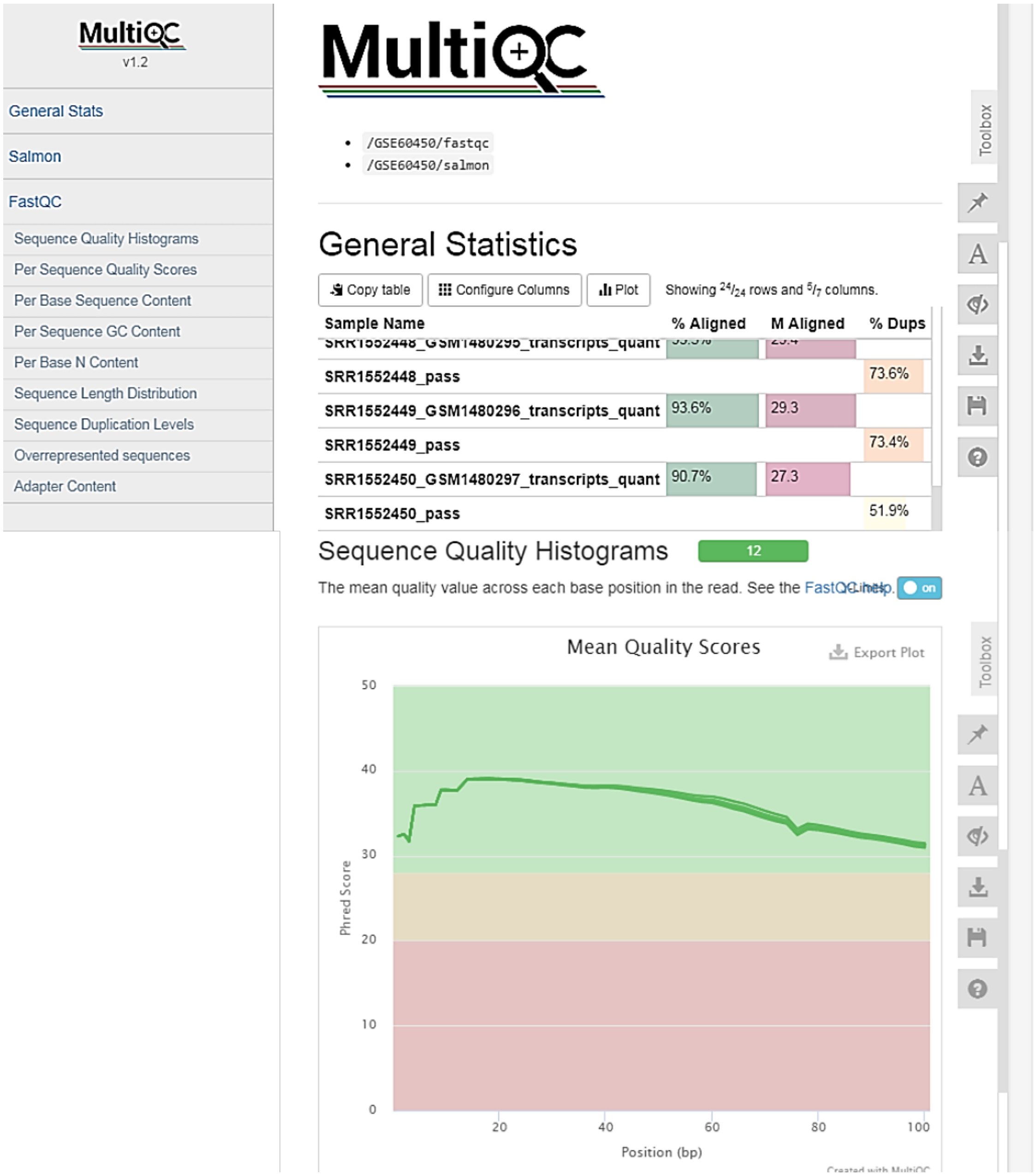

Supplementary Figure S7. MultiQC report. Both the *FastQC* and *Salmon* alignment reports are available for each of the samples. Besides the whole HTML report, all the tables and figures and individually downloadable.

Visualization

This section provides access to four different types of interactive exploratory plots. These plots are important in order to uncover underlying relationship of the samples and gain deeper insight of the data structure. We leverage several state-of-the-art R and Bioconductor packages for this purpose.

Correlation and density plot

Sample-wise Pearson correlation heatmap and density plot are generated using *Plotly*. User can hover over the plots to see expression values or zoom in to any specific area and double click to zoom out. Group wise annotation is available for correlation heatmap. Distribution of the data on the $log_2(Counts\ per\ million)$ scale is shown in the density plot.

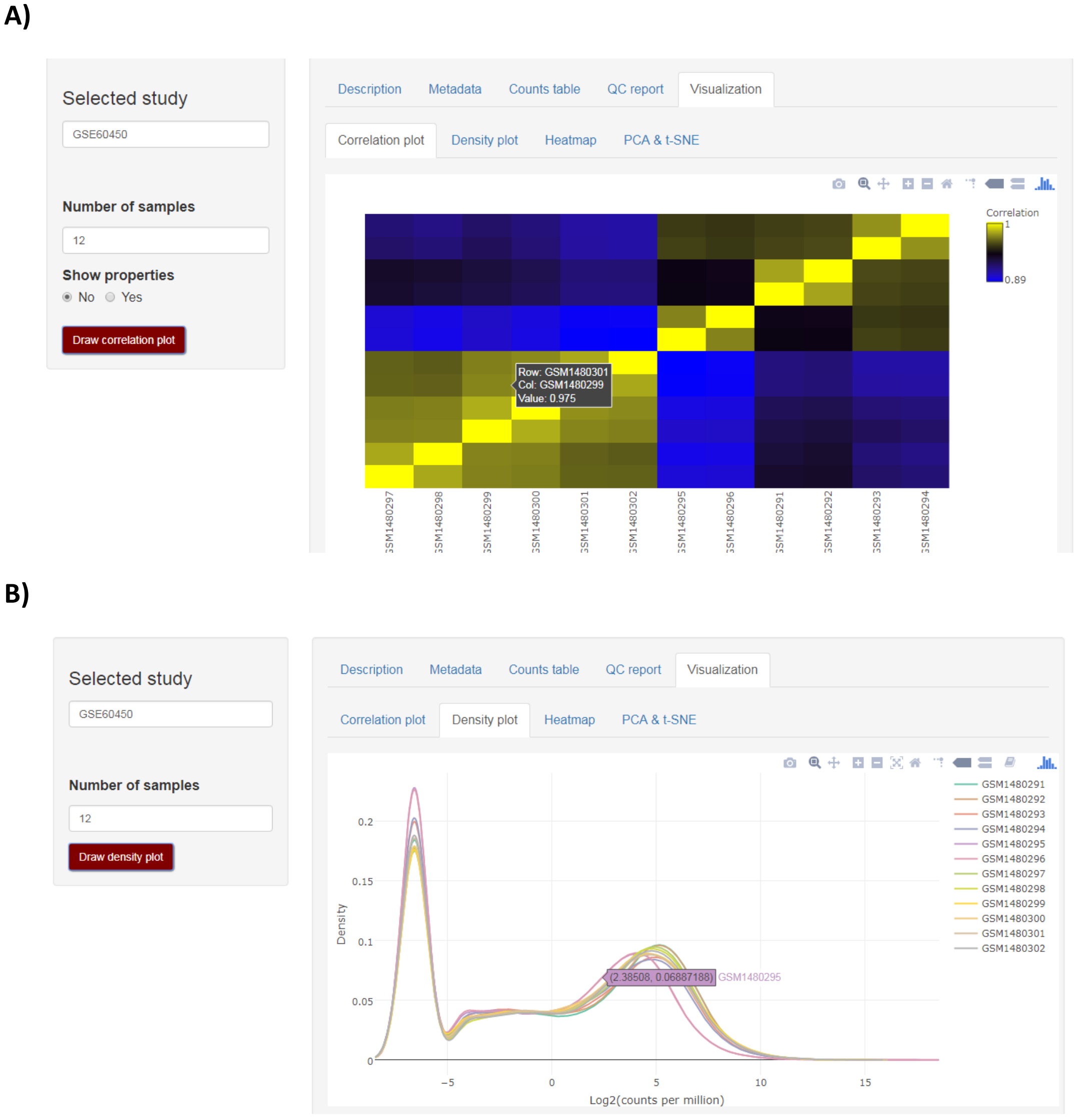

Supplementary Figure S8. Correlation and density plot tab panel. (**A**) Pearson correlation heatmap shows high correlations within each cell line which indicate high quality of transcriptional profiles. (**B**) Density plot of each sample based on counts per million on log2 scale. User can select any number of samples or groups. User can deselect any sample from the legend in the right side by just clicking on the sample names.

Heatmap

Heatmap is displayed based on the top most highly variable genes (sorted by median absolute deviation values of $log_2(Counts\ per\ million)$ and data is centered to the mean) in this section. We use Bioconductor packages *ComplexHeatmap* and R package *iheatmapr* for static and interactive heatmaps respectively. User can select either Pearson correlation, Euclidean distance, or group by properties option for hierarchical clustering of both genes and samples. User can also pick any number of highly variable genes. Both the plots and heatmap data are downloadable.

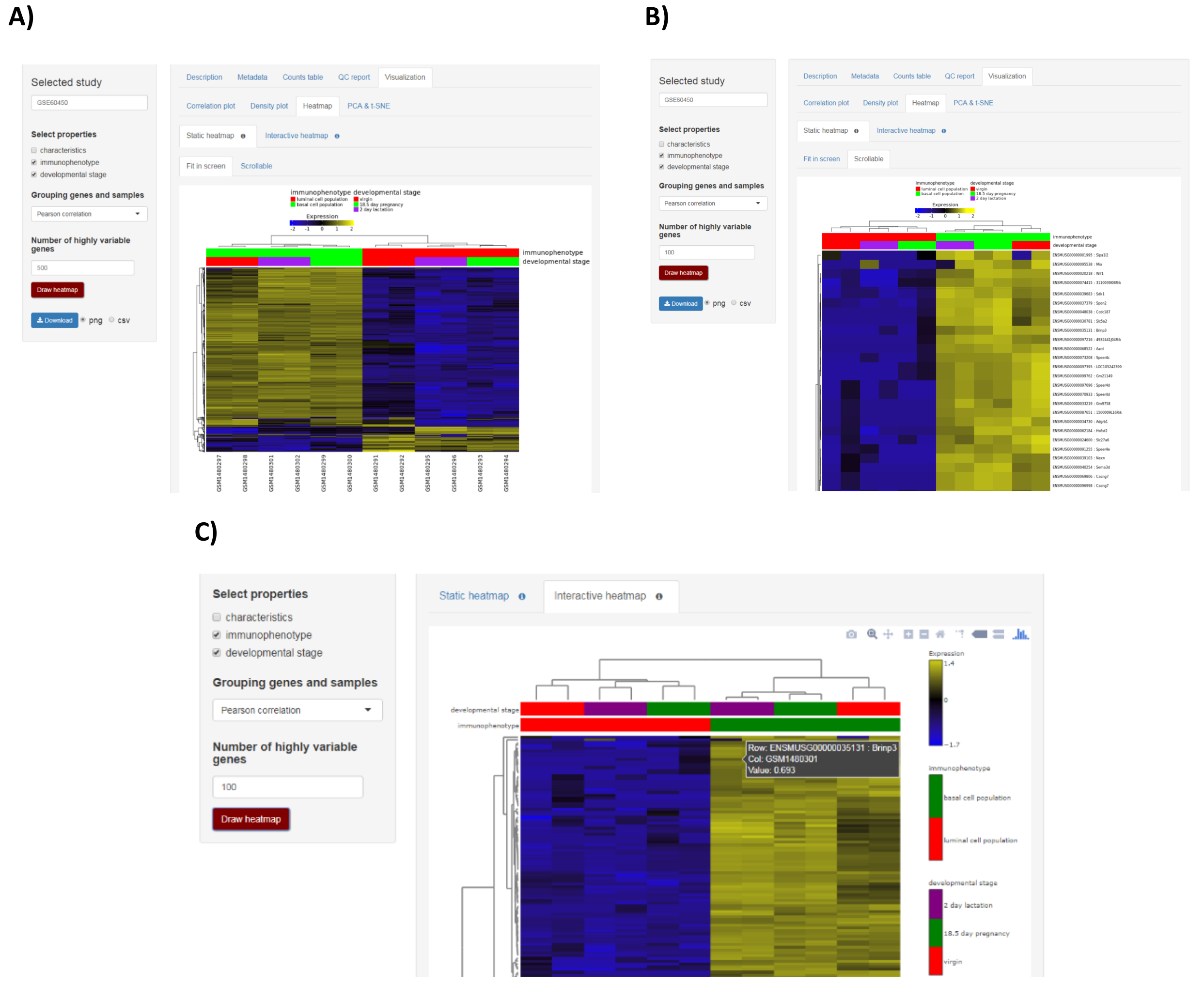

Supplementary Figure S9. Heatmap tab panel. (**A**) Static heatmap (Fit in screen) shows a complete picture that fits to the window without the gene symbols. (**B**) Static heatmap (Scrollable) shows the gene symbol. (C) Interactive heatmap provides the option to see the values and gene symbols while hovering over the heatmap as well as zooming in and out. User can select any area to zoom in and double-click to zoom out.

Principal component analysis (PCA) and t-distributed stochastic neighboring embedding (t-SNE) plots

GREIN provides the options for visualizing data in reduced dimension using both linear and non-linear approaches. PCA and t-SNE plots are available in both two and three-dimensional plane. User can subset the data and mouse hover on each data points to see the labels.

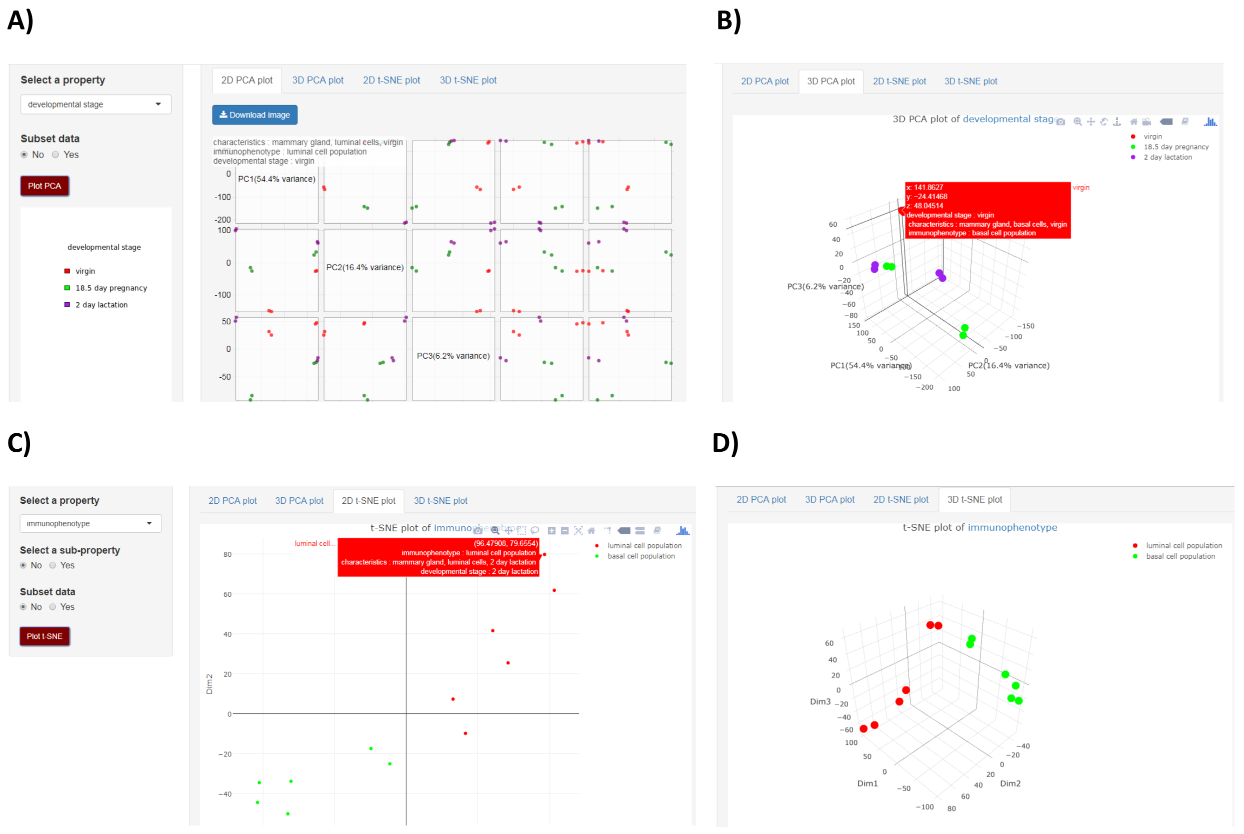

Supplementary Figure S10. PCA and t-SNE tab panel. (**A**) Scatter plot matrix of the first five principal components in $\boldsymbol{log_2(Counts\ per\ million)}$ scale. User can mouse hover to see the sample labels or make a square box on single or multiple points to see the location of these points in the graph. (**B**) Three-dimensional PCA plot provides more visual flexibility of the principal components on a 3-D plane. (**C**) Two-dimensional t-SNE plot can be visualized using sub-properties or sub-set of data. (**D**) 3-D t-SNE plot visualizes t-SNE embedding in a three dimension for better understanding.

Analyze dataset

The **'Analyze dataset'** tab at the very top tab panel consists of **'Power analysis'** and **'Create a signature'** tabs.

Power analysis

This section is dedicated to assist users in power analysis which is an essential step in designing an RNA-seq experiment with a goal to achieve the desired power to detect differentially expressed genes. This section is comprised of three sub-sections: metadata, power curve and detectability of genes. User will have to select two groups for power analysis.

Power curve

We use Bioconductor package *RNASeqPower* to calculate power using the following parameters:

1. Biological coefficient of variation calculated as the squared root of the common dispersion (We use Bioconductor package *edgeR* to calculate common dispersion).
2. Number of samples in each group.
3. Fold change as the effect size. The default value is 2.
4. Level of significance or alpha. The default value is 0.01.
5. Average sequencing depth in million. The default is calculated as the average column sums in million.

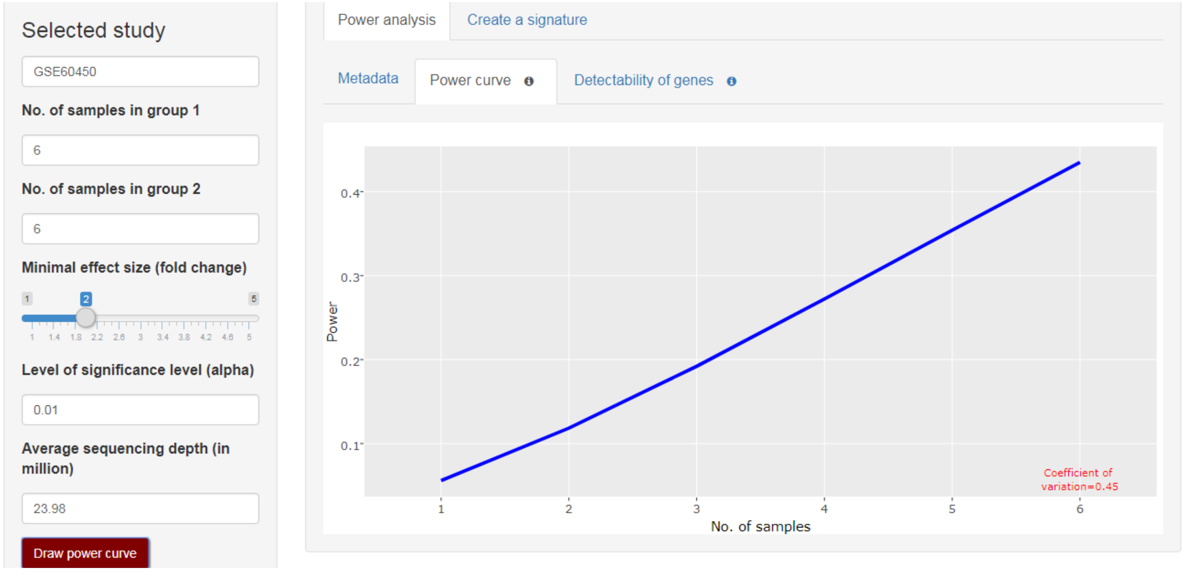

Supplementary Figure S11. Power curve tab panel. This plot is based on a single gene.

Detectability of genes

The plot of biological coefficient of variation (BCOV) vs. average $log_2(Counts\ per\ million)$ gives an idea regarding the detectability of each of the genes as differentially expressed based on the selected groups. User can modify the parameters as per their interest. Also, user can search for a gene to see the power of the gene or clicking on the points will display the values in a table below the plot.

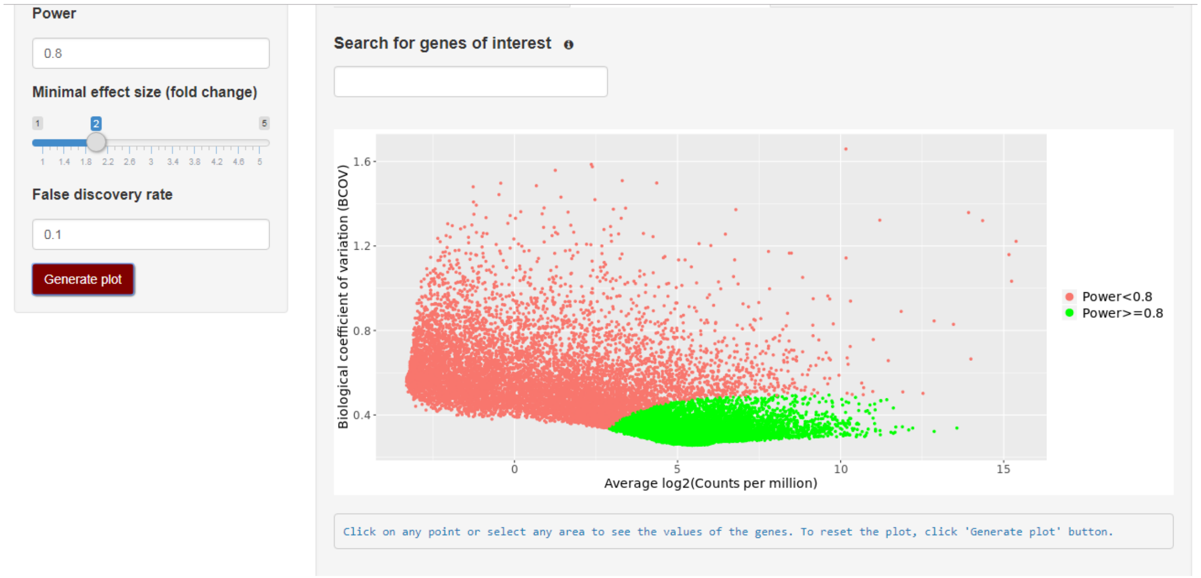

Supplementary Figure S12. Detectability of genes tab panel. User can search for any gene symbol to see its detectability power.

Create a signature

Generating differential expression signature is one of the most important segments of GREIN. This section begins with selecting a variable of interest to test for differential expression between the groups of this variable. We would like to see transcriptional changes between lactating and pregnant samples from the basal population only. So, we select **'developmental stage'** as our factor of interest, select **'2 day lactation'** in the experimental group and **'18.5 day pregnancy'** in the control group. Depending on the number of available properties and levels, two different types of comparisons are available: two group without covariate and two group with covariate. Then we select **'Yes'** for the **'Subset samples'** which provides the option to select basal population only. User can see the selected groups in the **'Metadata'** table (Supplementary Figure S13). The variable **'Selected groups'** in this table is created on the fly based on the selected groups. A signature table will be generated once the **'Generate signature'** button is clicked (Supplementary Figure S14). The analysis pipeline starts by filtering genes with very low counts. Genes that have counts per million (CPM) values of more than 0 in at least the minimum number of samples in any of the comparison groups are kept for the downstream analysis. We apply trimmed mean of M values (TMM) for normalizing libraries which is a built-in normalization method in *edgeR*. A design matrix is constructed based on the selected variable and groups. We use gene-wise negative binomial generalized linear models with quasi-likelihood tests and gene-wise exact tests from Bioconductor package *edgeR* to calculate differential expression between groups with and without covariates respectively. P-values are adjusted for multiple testing correction using Benjamini-Hochberg method. A gene is considered up-regulated in the **'2 day lactation'** group if $log_2(fold\ change)$ (logFC) is positive and a gene is down-regulated if $log_2(fold\ change)$ is negative.

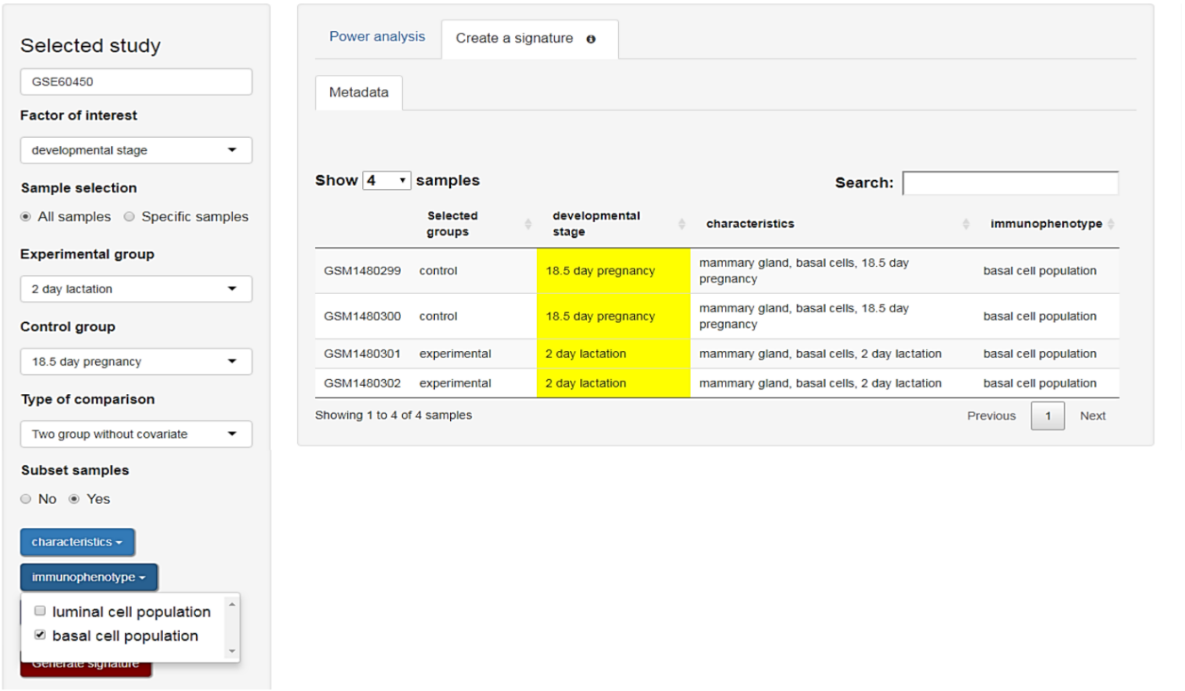

Supplementary Figure S13. Metadata table in the 'Create a signature' tab panel. User can subset the data or select specific samples for each group.

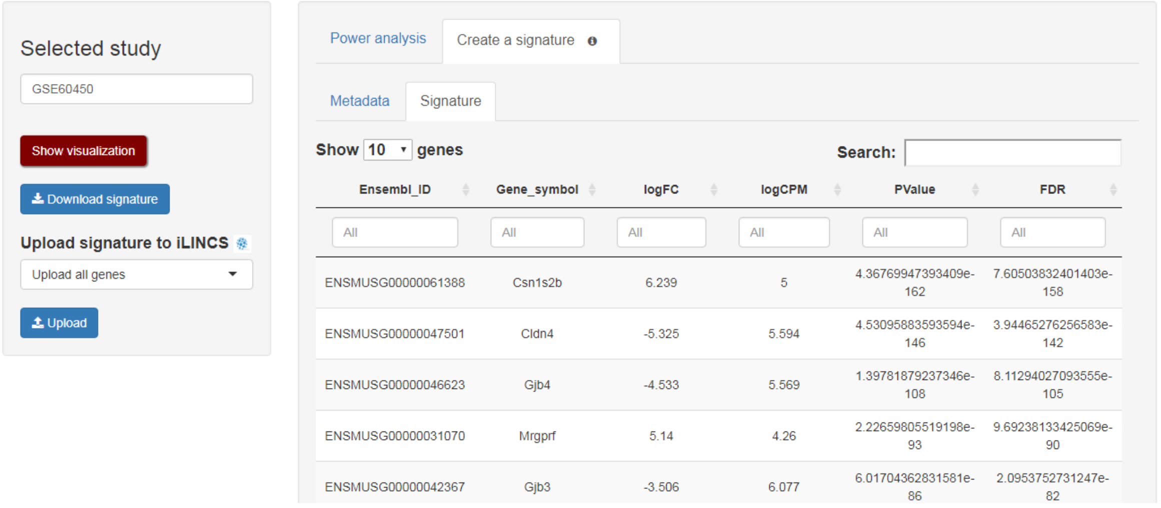

Supplementary Figure S14. Signature table in the 'Create a signature' tab panel. The table is downloadable is CSV format. User can also search for genes in the top or column search boxes.

There are three separate buttons in this tab panel: **'Show visualization'**, **'Download signature'**, and **'Upload'** which uploads list of genes including all, up-regulated, down-regulated, differentially expressed (DE), and a set of DE and not DE but detectable (NDE&DT) genes to iLINCS. A pop-up window will

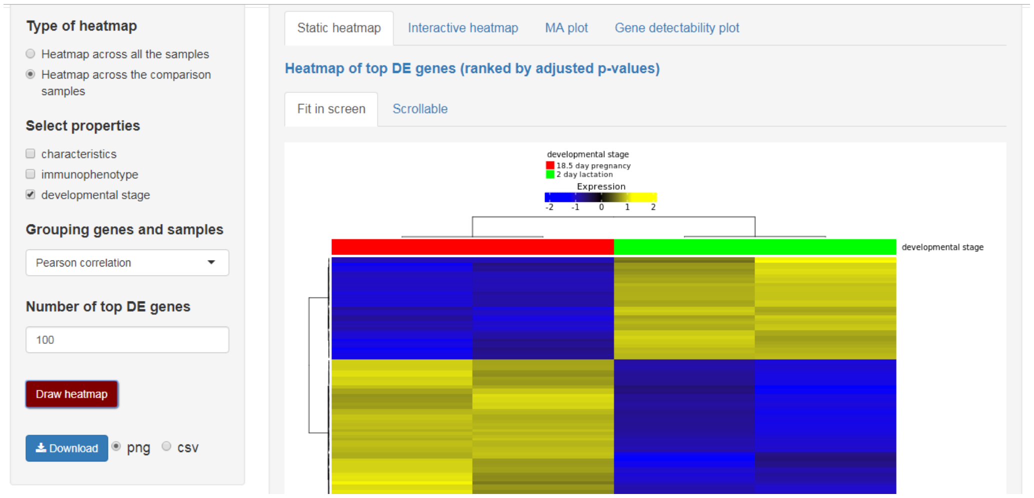

Supplementary Figure S15. Heatmap of top 100 differentially expressed genes. User can also visualize across all the samples.

appear if **'Show visualization'** button is clicked. Heatmap, MA plot, and gene detectability plots are included in this section based on the top most differentially expressed genes. The heatmap shows the change in relative expression of the genes. User can select to show the heatmap across all the samples or the comparison samples only (See Supplementary Figure S15). The MA plot visualizes the relationship between effect size and expression of the genes in log scale (See Supplementary Figure S16).

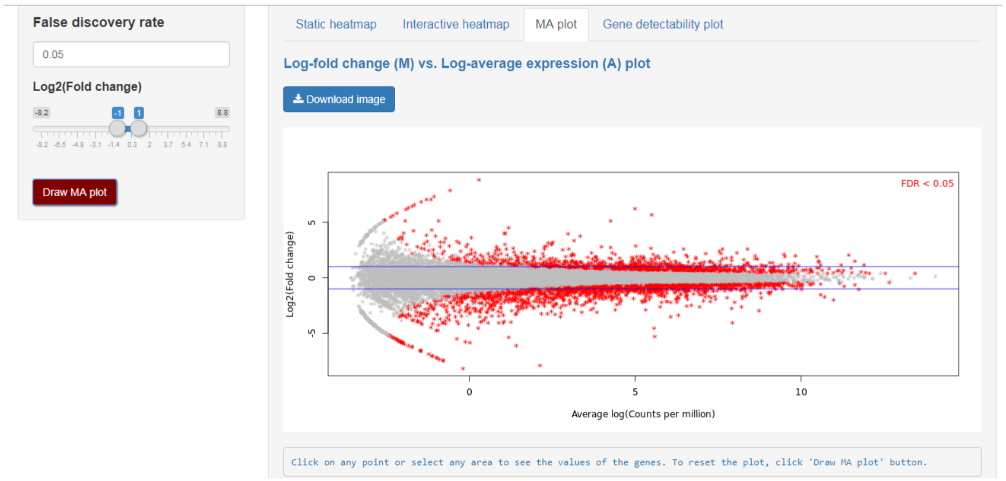

Supplementary Figure S16. MA plot with FDR cutoff. User can click on any point to see the corresponding values. The blue line is the default log fold change cut off.

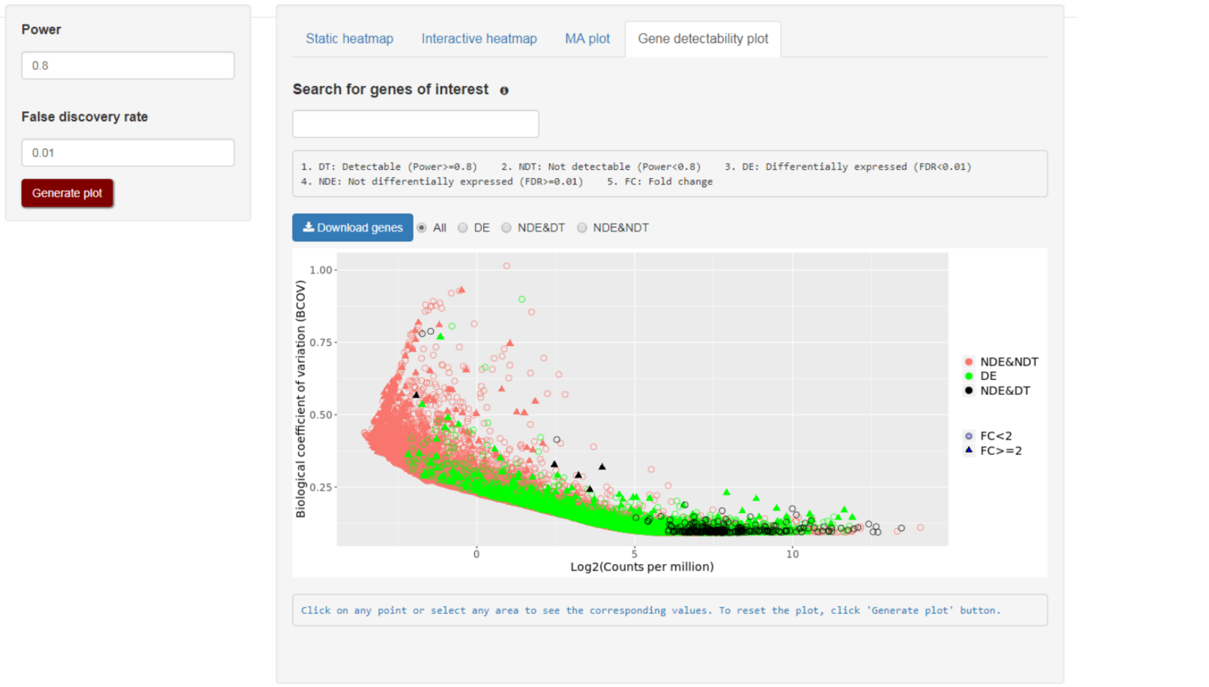

Supplementary Figure S17. Gene detectability plot. User can select genes based on the cutoff or search for genes of interest to see their power. User can also select any specific area in the plot to see the signature table of the selected genes.

Gene detectability plot shows the effect of BCOV or average depth on the power and identifies genes that might act as false negatives (See Supplementary Figure S17). User can download signature data based on their selection (DE, NDE&DT, or NDE&NDT).

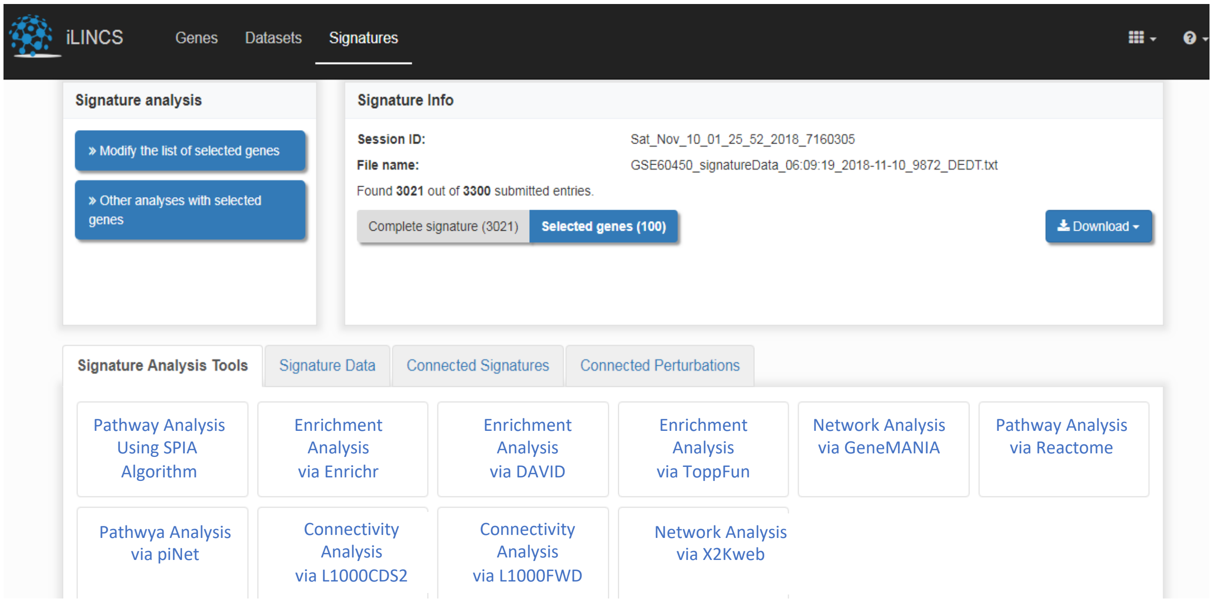

Supplementary Figure S18. Uploaded signature to iLINCS portal. User can further modify the list of genes.

The **'Download signature'** button in iLINCS (See Supplementary Figure S14) lets user download the signature data table in CSV format. Finally, pressing **'upload'** (See Supplementary Figure S14) button will open the iLINCS (http://www.ilincs.org) portal (See Supplementary Figure S18). Integrative LINCS or iLINCS is an integrative and user-friendly web platform with a number of tools for analysis of LINCS and non-LINCS data and signatures. User can upload or select a signature, conduct enrichment analysis, find concordant signatures, and analyze them to identify meaningful biological pathways. It is a part of NIH LINCS (http://www.lincsproject.org/) Common Fund program.

References

1. Hart, S. N., Therneau, T. M., Zhang, Y., Poland, G. A. & Kocher, J.-P. Calculating Sample Size Estimates for RNA Sequencing Data. *J. Comput. Biol.* **20**, 970-978, doi:10.1089/cmb.2012.0283 (2013).
2. Chang, W., Cheng, J., Allaire, J. J., Xie, Y. & McPherson, J. Shiny: web application framework for R. *R package version 0.11* **1**, 106 (2015).
3. Davis, S. & Meltzer, P. S. GEOquery: a bridge between the Gene Expression Omnibus (GEO) and BioConductor. *Bioinformatics* **23**, 1846-1847, doi:10.1093/bioinformatics/btm254 (2007).
4. Robinson, M. D., McCarthy, D. J. & Smyth, G. K. edgeR: a Bioconductor package for differential expression analysis of digital gene expression data. *Bioinformatics* **26**, 139-140, doi:10.1093/bioinformatics/btp616 (2010).
5. Sievert, C. *et al.* plotly: Create Interactive Web Graphics via 'plotly. js'. *R package version 4.7. 1* (2017).
6. Gu, Z., Eils, R. & Schlesner, M. Complex heatmaps reveal patterns and correlations in multidimensional genomic data. *Bioinformatics* **32**, 2847-2849 (2016).
7. Schep, A. N. & Kummerfeld, S. K. iheatmapr: interactive complex heatmaps in R. *J Open Source Software* **2**, 359 (2017).
8. Adler, D., Murdoch, D., Nenadic, O. & Urbanek, S. rgl: 3D visualization device system (OpenGL). *R package version 0.75* (2007).
9. Krijthe, J. H. Rtsne: T-distributed stochastic neighbor embedding using Barnes-Hut implementation. *R package version 0.13* (2015).
10. Aspera Connect. https://www.asperasoft.com (accessed, 5 October 2018).
11. NCBI SRA toolkit. http://trace.ncbi.nlm.nih.gov/Traces/sra/sra.cgi?view=software (accessed, 5 October 2018).
12. Andrews, S. FastQC: a quality control tool for high throughput sequence data. http://www.bioinformatics.babraham.ac.uk/projects/fastqc (2010).
13. Bolger, A. M., Lohse, M. & Usadel, B. Trimmomatic: a flexible trimmer for Illumina sequence data. *Bioinformatics* **30**, 2114-2120, doi:10.1093/bioinformatics/btu170 (2014).
14. Patro, R., Duggal, G., Love, M. I., Irizarry, R. A. & Kingsford, C. Salmon provides fast and bias-aware quantification of transcript expression. *Nat. Methods* **14**, 417, doi:10.1038/nmeth.4197 (2017).
15. Soneson, C., Love, M. I. & Robinson, M. D. Differential analyses for RNA-seq: transcript-level estimates improve gene-level inferences. *F1000Res.* **4**, 1521, doi:10.12688/f1000research.7563.2 (2015).

16. Ewels, P., Magnusson, M., Lundin, S. & Käller, M. MultiQC: summarize analysis results for multiple tools and samples in a single report. *Bioinformatics* **32**, 3047-3048, doi:10.1093/bioinformatics/btw354 (2016).
17. Bernstein, M. N., Doan, A. & Dewey, C. N. MetaSRA: normalized human sample-specific metadata for the Sequence Read Archive. *Bioinformatics* **33**, 2914-2923, doi:10.1093/bioinformatics/btx334 (2017).
18. Fu, N. Y. *et al.* EGF-mediated induction of Mcl-1 at the switch to lactation is essential for alveolar cell survival. *Nat. Cell Biol.* **17**, 365 (2015).
